# Oxygen consumption is depressed in patients with lactic acidosis due to biguanide intoxication

**DOI:** 10.1186/cc8885

**Published:** 2010-02-19

**Authors:** Alessandro Protti, Riccarda Russo, Paola Tagliabue, Sarah Vecchio, Mervyn Singer, Alain Rudiger, Giuseppe Foti, Anna Rossi, Giovanni Mistraletti, Luciano Gattinoni

**Affiliations:** 1 Fondazione IRCCS Ospedale Maggiore Policlinico, Mangiagalli e Regina Elena di Milano, Università degli Studi di Milano, Via F. Sforza 35, 20122 Milan, Italy; 2 Ospedale San Gerardo Nuovo dei Tintori, Università di Milano-Bicocca, Piazza dell'Ateneo Nuovo 1, 20126, Milan, Italy; 3 Centro Nazionale di Informazione Tossicologica, Fondazione IRCCS Salvatore Maugeri, Via Maugeri 10, 27100 Pavia, Italy; 4 Bloomsbury Institute of Intensive Care Medicine, University College London, 5 University Street, London WC1E 6JF, UK; 5 University Hospital Zurich, Rämistrasse 100, 8091 Zürich, Switzerland; 6 Ospedale Niguarda Ca' Granda, Piazza Ospedale Maggiore 3, 20162 Milan, Italy; 7 Ospedale San Paolo, Università degli Studi di Milano, Via A. Di Rudiní 8, 20142 Milan, Italy

## Abstract

**Introduction:**

Lactic acidosis can develop during biguanide (metformin and phenformin) intoxication, possibly as a consequence of mitochondrial dysfunction. To verify this hypothesis, we investigated whether body oxygen consumption (VO_2_), that primarily depends on mitochondrial respiration, is depressed in patients with biguanide intoxication.

**Methods:**

Multicentre retrospective analysis of data collected from 24 patients with lactic acidosis (pH 6.93 ± 0.20; lactate 18 ± 6 mM at hospital admission) due to metformin (n = 23) or phenformin (n = 1) intoxication. In 11 patients, VO_2 _was computed as the product of simultaneously recorded arterio-venous difference in O_2 _content [C(a-v)O_2_] and cardiac index (CI). In 13 additional cases, C(a-v)O_2_, but not CI, was available.

**Results:**

On day 1, VO_2 _was markedly depressed (67 ± 28 ml/min/m^2^) despite a normal CI (3.4 ± 1.2 L/min/m^2^). C(a-v)O_2 _was abnormally low in both patients either with (2.0 ± 1.0 ml O_2_/100 ml) or without (2.5 ± 1.1 ml O_2_/100 ml) CI (and VO_2_) monitoring. Clearance of the accumulated drug was associated with the resolution of lactic acidosis and a parallel increase in VO_2 _(*P *< 0.001) and C(a-v)O_2 _(*P *< 0.05). Plasma lactate and VO_2 _were inversely correlated (R^2 ^0.43; *P *< 0.001, n = 32).

**Conclusions:**

VO_2 _is abnormally low in patients with lactic acidosis due to biguanide intoxication. This finding is in line with the hypothesis of inhibited mitochondrial respiration and consequent hyperlactatemia.

## Introduction

Metformin and phenformin are oral anti-diabetic drugs of the biguanide class. Metformin is the first-line drug of choice for the treatment of adults with type 2 diabetes [1]. It is the 10^th ^most frequently prescribed generic drug in the USA (>40 million prescriptions in 2008) and is currently used by almost one-third of diabetic patients in Italy [2,3]. Phenformin is no longer on sale in many countries, but is still available in Italy.

Lactic acidosis can develop in patients taking metformin or phenformin, especially when renal failure leads to drug accumulation [4-6]. According to the American Association of Poison Control Centers, metformin was implicated in 19 fatalities in the USA in 2007 [7]. Thirty cases of biguanide intoxication have been reported over the past two years to the Poison Control Centre of Pavia, Italy, resulting in 10 deaths (Dr Sarah Vecchio, unpublished data). The progressive increase in metformin use (20% rise in prescriptions between 2006 and 2008 in the USA) may result in a parallel increase in the incidence of associated lactic acidosis [2,8].

The pathogenesis of biguanide-associated lactic acidosis remains unclear, especially when it develops in the absence of other major risk factors such as hypoxia, tissue hypoperfusion, or liver failure (biguanide-induced lactic acidosis). Hyperlactatemia is classically attributed to an impaired lactate clearance, secondary to an exaggerated inhibition of hepatic gluconeogenesis [9] but may also depend on an increased lactate production by the liver [10] or the intestine [11].

Biguanide drugs mainly exert their therapeutic effect by impairing hepatocyte mitochondrial respiration [12,13]. Recent observations have suggested that metformin, similarly to phenformin, might also inhibit mitochondrial respiration in tissues other than the liver [14-16]. Mitochondria produce energy while consuming oxygen (O_2_) and releasing carbon dioxide (CO_2_) and heat. When O_2 _provision or utilization are compromised, cellular energy production can partly rely on the extra-mitochondrial anaerobic lactate generation, that is associated with metabolic acidosis. As mitochondrial respiration normally accounts for more than 90% of whole body O_2 _utilization and CO_2 _release, any defect in mitochondrial metabolism will decrease systemic O_2 _consumption and CO_2 _production.

We hypothesize that inhibition of mitochondrial respiration is responsible for the development of lactic acidosis during metformin or phenformin intoxication. If our hypothesis is correct, respiration should be abnormally low regardless of any change in systemic O_2 _delivery. The aim of this study is to investigate global O_2 _consumption (and CO_2 _production) in patients with lactic acidosis due to biguanide intoxication.

## Materials and methods

We reviewed the data sheets of patients admitted to 12 intensive care units and 1 nephrology unit of 11 hospitals from January 2005 to June 2009, with a discharge diagnosis of lactic acidosis due to biguanide intoxication. Patients with a concomitant primary diagnosis of septic or cardiogenic shock or liver failure were excluded. Lactic acidosis was defined as pH less than 7.30 and plasma lactate more than 5 mM. Only patients with central or mixed venous O_2 _saturation monitoring were included.

We calculated the arterio-venous difference in O_2 _content [C(a-v)O_2_] as:

where CaO_2 _and CvO_2 _are arterial and venous blood O_2 _content, respectively, Hb is blood hemoglobin concentration, SaO_2 _is arterial O_2 _saturation, SvO_2 _is O_2 _saturation of blood taken from the superior vena cava or the pulmonary artery (collectively indicated as central venous blood) and PaO_2 _and PvO_2 _are the arterial and central venous O_2 _tensions.

Oxygen extraction index (OEI) was defined as:

and expressed as a percentage. The veno-arterial difference in CO_2 _content [C(v-a)CO_2_] was calculated according to Douglas and colleagues [17]. In patients with cardiac index (CI) monitoring, we calculated whole body O_2 _delivery (DO_2_) as CI × CaO_2 _and O_2 _consumption (VO_2_) as CI × C(a-v)O_2_, with CI computed as cardiac output divided by estimated body surface area. Carbon dioxide production (VCO_2_) was calculated as CI × C(v-a)CO_2_.

The severity of illness was initially expressed by the Simplified Acute Physiology Score (SAPS) II [18] and then monitored using the Sequential Organ Failure Assessment (SOFA) score [19]. The cardiovascular SOFA score was used to describe catecholamine requirements. Sedation was evaluated using the Richmond Agitation Sedation Scale (RASS) [20]. Heart rate, body temperature and need for mechanical ventilation were also recorded. Analysis was restricted to the first four days following admission, or until discharge or death if any of these occurred earlier.

The local Ethics Committee of the coordinating Centre (Fondazione IRCCS Ospedale Maggiore Policlinico, Mangiagalli e Regina Elena di Milano, Italy) was informed of the ongoing retrospective analysis and did not require any specific informed consent.

### Statistical analysis

Results are presented as mean ± standard deviation or median and interquartile range, based on data distribution (Kolmogorov-Smirnov test). The relation between serum metformin levels and other variables was assessed using linear regression analysis and expressed as R^2^. Severity of illness at admission of patients with or without CI monitoring was compared using the Student's *t*-test. The remainder of the analyses were performed on data averaged on a daily basis. Changes occurring over time were investigated using parametric or non-parametric one-way repeated-measures analysis of variance. *Post-hoc *comparisons were performed using Bonferroni or Dunn's test, considering day 1 as baseline. The relation between the arterio-venous difference in O_2 _content and the veno-arterial difference in CO_2 _content was calculated using linear regression. The relation between systemic O_2 _consumption and other variables was investigated using linear (arterial pH) or non-linear (body temperature and plasma lactate) regression. The chi-squared test was used to assess whether the proportion of patients requiring mechanical ventilation changed over time. Analysis was performed using Sigma Stat version 3.1.1 (Jandel Scientific Software; San Jose, CA, USA). A two-sided *P *value less than 0.05 was considered as statistically significant.

## Results

We identified 24 diabetic patients admitted to the intensive care (n = 22) or nephrology (n = 2) units with lactic acidosis attributed to either metformin (n = 23) or phenformin (n = 1) intoxication (Table 1). Seventeen (71%) were females and the mean age of all patients was 66 ± 9 years.

**Table 1:** Main characteristics of the study population

Id	Intoxicant	Serum drug level (μg/ml)	Creatinine (mg/dl)	pH	Lactate (mM)	Monitoring	SAPS II	ICU outcome
1	Metformin	70	6.4	7.21	22	CI;	58	S
2	Metformin	63	12.4	6.95	33	CI;	53	S
3	Metformin	NA	10.8	6.76	21	CI;	61	S
4	Metformin	NA	15.2	7.06	18	CI;	51	S
5	Metformin	NA	9.0	6.63	21	CI;	55	S
6	Metformin	NA	10.3	6.82	21	CI; ScvO_2_	87	S
7	Metformin	NA	10.8	6.70	24	CI; ScvO_2_	74	S
8	Metformin	61	1.9	7.27	10	CI;	83	NS
9	Metformin	NA	13.2	6.79	21	CI; ScvO_2_	63	S
10	Metformin	NA	4.7	7.13	19	CI; ScvO_2_	66	S
11	Metformin	53	4.5	<6.80	16	CI;	87	NS
12	Metformin	65	8.4	6.76	22	ScvO_2_	43	S
13	Phenformin	480^§^	9.5	6.91	13	ScvO_2_	59	S
14	Metformin	100	5.8	7.26	10	ScvO_2_	58	S
15	Metformin	63	4.2	6.89	18	ScvO_2_	53	NS
16	Metformin	NA	13.0	6.93	17	ScvO_2_	67	S
17	Metformin	19^†^	9.9	6.62	19	ScvO_2_	62	S
18	Metformin	NA	6.1	<6.80	24	ScvO_2_	70	NS
19	Metformin	100	7.6	6.87	16	ScvO_2_	45	S
20	Metformin	25^†^	9.3	6.81	15	ScvO_2_	44	S
21*	Metformin	70	4.8	7.22	11	ScvO_2_	66	S
22*	Metformin	44	10.0	6.93	14	ScvO_2_	55	S
23	Metformin	NA	13.8	7.21	6	ScvO_2_	39	S
24	Metformin	NA	7.1	6.93	17	ScvO_2_	65	NS

The first available serum drug concentration (^§ ^phenformin in ng/ml;^† ^blood sample obtained with ongoing renal replacement therapy), creatinine level, arterial blood pH and plasma lactate level, available data (CI, cardiac index; ScvO_2_, central venous oxymetry; , mixed venous oxymetry), severity of the disease (expressed as Simplified Acute Physiology Score (SAPS) II score) and outcome (S = survivor; NS = non survivor) are reported. Target values in patients on metformin or phenformin are less than 4 μg/ml and less than 140 ng/ml, respectively. ICU, intensive care unit; NA, not available; * patients admitted to the Nephrology Unit.

Lactic acidosis on hospital admission was always severe, with an arterial pH of 6.93 ± 0.20 and lactate of 18 ± 6 mM. Blood glucose level was 118 ± 78 mg/dl, with severe hypoglycemia (<40 mg/dl) being present in 3 patients. Liver function tests were usually normal, with alanine aminotransferase 66 ± 78 IU/L, total bilirubin 0.4 ± 0.2 mg/dl, albumin 33 ± 6 g/L, and prothrombin time (expressed as international normalized ratio) 1.2 ± 0.3 (excluding two patients on warfarin). Left ventricular ejection fraction, investigated in seven patients by echocardiography, was always normal (≥ 50%).

Intoxication was always accidental and associated with renal failure (creatinine 8.7 ± 3.5 mg/dl, urea 171 ± 70 mg/dl and oligo-anuria) and continued drug intake. Factors potentially implicated in the development of renal failure were dehydration (a history of several days' vomiting and/or diarrhea was reported in 75% of the cases), urinary tract infection (29%) and chronic renal dysfunction (21%).

Whenever measured, serum drug concentration on day 1 was always well above safe limits (metformin 61 ± 25 vs. <4 μg/ml, n = 12; phenformin 480 vs. <140 ng/ml, n = 1). Metformin levels, measured at different time points in 10 patients, were positively correlated with those of creatinine (R^2 ^= 0.34; *P *< 0.001, n = 29) and lactate (R^2 ^= 0.49; *P *< 0.001, n = 29) and inversely correlated with arterial pH (R^2 ^= 0.68; *P *< 0.001, n = 29).

Treatment included the use of mechanical ventilation (n = 16), catecholamines (n = 21) and renal replacement therapy (n = 21). The first day SAPS II score was 61 ± 13, corresponding to an expected mortality of approximately 70%. Observed mortality was 21%.

Central venous O_2 _saturation was monitored through a central venous (n = 17) or pulmonary artery (n = 7) catheter. Blood gases were always measured at 37°C. In 11 patients, CI was also measured, using the PiCCO system (n = 2), transesophageal Doppler ultrasonography (n = 2) or pulmonary artery catheter thermodilution (n = 7). Patients with CI monitoring had a higher SAPS II (67 ± 14 vs. 56 ± 10; *P *< 0.05) and SOFA (12 ± 3 vs. 9 ± 2; *P *< 0.05) scores on admission.

**Figure 1:**
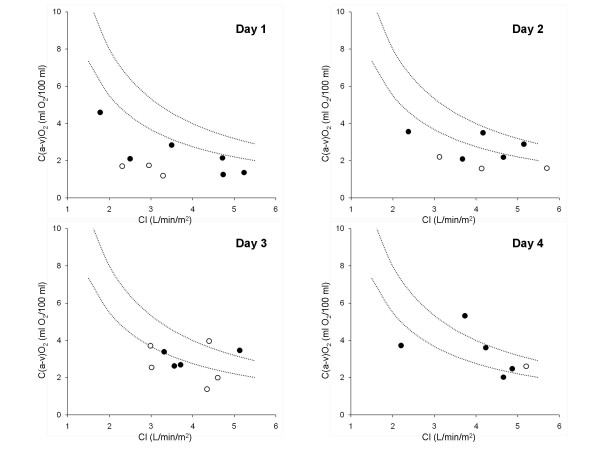
**Relation between cardiac index and arterio-venous difference in oxygen content in biguanide-intoxicated patients**. Cardiac index (CI) and arterio-venous difference in oxygen content [C(a-v)O_2_] recorded during the first 4 days of admission from 11 biguanide-intoxicated patients. Each circle refers to individual data averaged on a daily basis. The arterio-venous difference in oxygen content was computed from either mixed (black circles) or central (white circles) venous oxygen saturation. Dotted lines refer to the lower and upper limits of normal systemic oxygen consumption (110 to 160 ml/min/m^2^). Circles that are located under the lower dotted line indicate an arterio-venous difference in oxygen content (oxygen extraction) lower than expected if systemic oxygen consumption is normal.

**Figure 2:**
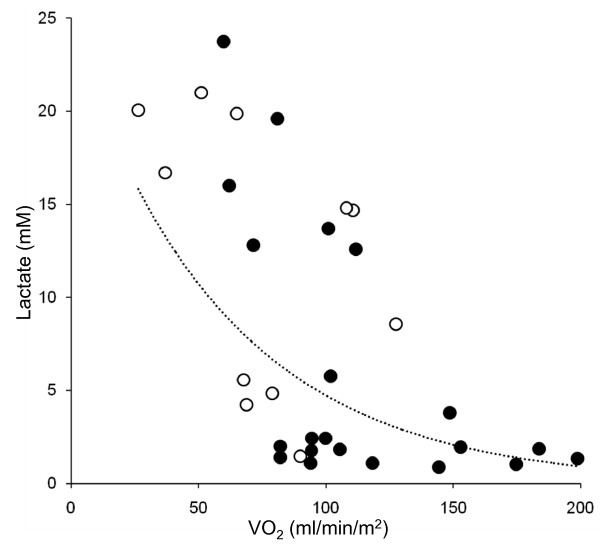
**Relation between systemic oxygen consumption and lactatemia in biguanide-intoxicated patients**. Systemic oxygen consumption (VO_2_), computed from either mixed (black circles) or central (white circles) venous oxygen saturation, inversely correlated with plasma lactate (R^2 ^= 0.43; *P *< 0.001; n = 32).

Main results are reported in Table 2 and Figures 1 and 2. Systemic O_2 _consumption, monitored in 11 patients, was abnormally low on day 1 and normalized within the next 48 to 72 hours (*P *< 0.001), paralleled by resolution of lactic acidosis (*P *< 0.001). As systemic O_2 _delivery did not significantly change compared with day 1, variations in whole body O_2 _consumption were reflected in equal changes in arterio-venous difference in O_2 _content and O_2 _extraction index and opposite changes in central venous O_2 _saturation (*P *< 0.001 for all). The difference in veno-arterial CO_2 _content was abnormally low on day 1 and progressively returned to normal (*P *< 0.05). Whole body CO_2 _production showed a similar, although not significant, trend, rising from 93 ± 24 (on day 1) to 115 ± 13 ml/min/m^2 ^(on day 4; n = 4). The arterio-venous difference in O_2 _content was positively associated with the veno-arterial difference in CO_2 _content (R^2 ^= 0.42; *P *= 0.001, n = 22). Systemic O_2 _consumption was positively associated with arterial pH (R^2 ^= 0.37; *P *< 0.001, n = 32) and body temperature (R^2 ^= 0.38; *P *< 0.001, n = 30) and inversely correlated with plasma lactate (R^2 ^= 0.43; *P *< 0.001, n = 32).

**Table 2:** Temporal changes observed in 11 biguanide-intoxicated patients with cardiac index and central venous oxygen saturation monitoring

	n	Day 1	Day 2	Day 3	Day 4	*P*
pH	11	7.03 (6.92-7.15)	7.35 (7.25-7.40)	7.44 (7.35-7.46)*	7.46 (7.44-7.47)*	<0.001
Lactate (mM)	11	17 (14-20)	5 (2-15)	2 (2-3)*	1 (1-3)*	<0.001
VO_2 _(ml/min/m^2^)	9	67 ± 28	99 ± 30*	116 ± 41*	129 ± 42*	<0.001
DO_2 _(ml/min/m^2^)	9	443 ± 167	572 ± 152	491 ± 95	430 ± 116	<0.01
CI (L/min/m^2^)	9	3.4 ± 1.2	4.4 ± 1.3	3.9 ± 0.8	3.4 ± 1.2	0.08
C(a-v)O_2_ (ml O_2_/100 ml)	10	2.0 ± 1.0	2.4 ± 0.8	2.9 ± 0.8*	3.8 ± 1.4*	<0.001
SvO_2 _(%)	10	83 ± 8	80 ± 6	75 ± 5*	70 ± 8*	<0.001
OEI (%)	10	13 (11-19)	16 (13-21)	23 (21-25)*	31 (23-34)*	<0.001
C(v-a)CO_2_ (ml CO_2_/100 ml)	7	2.2 ± 0.8	2.2 ± 0.8	3.9 ± 1.9	4.7 ± 1.2*	<0.05
RASS	11	-4 (-5--2)	-4 (-4--1)	-2 (-4-0)	-1 (-3-0)	0.06
On MV (%)	11	91	100	67	67	0.12
HR	11	103 ± 20	104 ± 8	99 ± 16	97 ± 21	0.74
SOFA	11	12 ± 3	10 ± 1*	9 ± 2*	10 ± 2*	<0.001
Catecholamine use (SOFA sub score)	11	4 (4-4)	4 (4-4)	4 (4-4)	3 (2-3)*	<0.001
BT (°C)	10	34.5 ± 2.2	36.6 ± 0.6*	36.8 ± 0.4*	36.7 ± 0.5*	<0.001

Results of repeated-measures analysis of variance and chi-squared test are reported in the right column. Data significantly different from day 1 on *post-hoc *comparison are indicated as *. n is the number of patients with each specific variable monitored on day 1.BT, body temperature; C(a-v)O_2_, arterio-venous difference in oxygen content; C(v-a)CO_2_, veno-arterial difference in carbon dioxide content; CI, cardiac index; DO_2_, systemic oxygen delivery; HR, heart rate; MV, mechanical ventilation; OEI, oxygen extraction index; RASS, Richmond Agitation Sedation Score; SOFA, Sequential Organ Failure Assessment; SvO_2_, central venous oxygen saturation; VO_2_, systemic oxygen consumption.

Major findings remained valid when the analysis was restricted to the 7 patients monitored with a pulmonary artery catheter. From day 1 to 4, lactate levels decreased from 16 (13 to 19) to 1 (1 to 2) mM (*P *< 0.01). Global O_2 _consumption increased (81 ± 21 vs. 129 ± 47 ml/min/m^2^; *P *= 0.01) despite no change in systemic O_2 _delivery (482 ± 180 vs. 441 ± 139 ml/min/m^2^; *P *= 0.10). The arterio-venous difference in O_2 _content (2.3 ± 1.2 vs. 3.9 ± 1.1 ml O_2_/100 ml; *P *= 0.001) and the O_2 _extraction index (17 ± 7 vs. 30 ± 6%; *P *< 0.001) augmented and the mixed venous O_2 _saturation accordingly decreased (81 ± 9 vs. 69 ± 6%; *P *= 0.001). The difference in veno-arterial CO_2 _content increased from 2.4 ± 0.7 to 4.6 ± 1.5 ml CO_2_/100 ml (*P *< 0.05). Systemic O_2 _consumption inversely correlated with plasma lactate (R^2 ^= 0.30; *P *= 0.01, n = 21).

In patients without CI monitoring, initial values and later changes in the other variables of interest closely resembled those observed in monitored patients (Table 3).

**Table 3:** Temporal changes observed in 13 biguanide-intoxicated patients with central venous oxygen saturation (but not cardiac index) monitoring

	n	Day 1	Day 2	Day 3	Day 4	*P*
pH	13	7.14 ± 0.17	7.36 ± 0.10*	7.45 ± 0.09*	7.43 ± 0.06*	<0.001
Lactate (mM)	13	12 ± 6	5 ± 8*	2 ± 1*	2 ± 1*	<0.001
C(a-v)O_2_ (ml O_2_/100 ml)	12	2.5 ± 1.1	3.1 ± 1.0	3.4 ± 0.8	4.2 ± 1.2*	<0.05
SvO_2 _(%)	12	79 ± 10	75 ± 10	73 ± 6*	66 ± 7*	0.01
OEI (%)	12	20 ± 10	24 ± 10	25 ± 7	33 ± 7*	0.01
C(v-a)CO_2_ (ml CO_2_/100 ml)	8	2.4 ± 1.6	2.8 ± 1.2	3.6 ± 0.9	5.5 ± 1.9	0.16
RASS	13	-1 (-4-0)	0 (-3-0)	0 (-1-0)	-1 (-3-0)	0.05
On MV (%)	13	31	42	27	38	0.89
HR	12	87 ± 17	88 ± 15	91 ± 14	88 ± 8	0.10
SOFA	13	9 ± 2	8 ± 3	6 ± 3*	7 ± 3*	<0.001
Catecholamine use (SOFA sub score)	13	3 ± 2	3 ± 2	2 ± 2*	2 ± 2*	<0.01
BT (°C)	10	35.8 (35.0-36.3)	36.8 (36.4-37.3)	37.0 (36.7-37.5)*	36.9 (36.6-37.4)	<0.05

Results of repeated-measures analysis of variance and chi-squared test are reported in the right column. Data significantly different from day 1 on *post-hoc *comparison are indicated as *. n is the number of patients with each specific variable monitored on day 1.

BT, body temperature; C(a-v)O_2_, arterio-venous difference in oxygen content; C(v-a)CO_2_, veno-arterial difference in carbon dioxide content; HR, heart rate; MV, mechanical ventilation; OEI, oxygen extraction index; RASS, Richmond Agitation Sedation Score; SOFA, Sequential Organ Failure Assessment; SvO_2_, central venous oxygen saturation.

Twelve patients had one or more simultaneous determinations of serum metformin levels and arterio-venous difference in O_2 _content; an inverse correlation was noted between these variables (R^2 ^= 0.20; *P *< 0.05, n = 22).

## Discussion

The present study demonstrates that whole body O_2 _consumption (and CO_2 _production) are abnormally low during biguanide-induced lactic acidosis and return to normal on recovery from drug intoxication.

Metformin is a safe drug when correctly prescribed [21]. Lactic acidosis can develop in cases of drug accumulation but is usually attributed to other concomitant precipitating factors. However, some reports suggest that metformin accumulation may cause lactic acidosis even in the absence of other obvious confounding variables [22]. According to discharge diagnosis, patients included in this present study suffered from lactic acidosis (better defined as hyperlactatemia with metabolic acidosis) mainly attributed to (documented or suspected) metformin or phenformin intoxication. None of the patients had any sign of acute liver or cardiac failure. Acute renal failure was invariably present at hospital admission, but could have hardly represented the sole cause of such a dramatic rise in blood lactate levels. Septic shock was never reported as the primary diagnosis. Sepsis may still have acted as a precipitating factor (gastroenteritis, urinary tract infection) but could not explain our present initial findings. Indeed, systemic O_2 _consumption is usually normal or even increased in critically ill septic patients, at least in the early phase [23,24].

The most common cause of lactic acidosis in critically ill patients is probably cellular hypoxia. When O_2 _delivery acutely decreases due to low cardiac output, anemia or hypoxemia, tissue O_2 _extraction rises in an attempt to preserve aerobic mitochondrial respiration. The arterio-venous difference in O_2 _content, that is the ratio between whole body O_2 _consumption and cardiac output, increases and central venous O_2 _saturation decreases. Oxygen consumption only starts to diminish when O_2 _delivery falls below a critical value; the blood lactate concentration then abruptly increases, indicating the development of anaerobic metabolism [25]. The veno-arterial difference in CO_2 _content, that depends on the ratio between CO_2 _production and cardiac output, may rise as well, mainly as a consequence of a reduced cardiac output.

Lactic acidosis can also develop under aerobic conditions, when O_2 _utilization is prevented by mitochondrial dysfunction, glycolysis is overly stimulated or lactate clearance is impaired [26-28]. Growing evidence, mainly derived from cell and animal studies, suggest that metformin and phenformin can actually interfere with mitochondrial respiration in a dose-dependent manner [10,12-14]. By interfering with mitochondrial respiration in the liver, they decrease gluconeogenesis (and lactate clearance) and may potentially increase glucose consumption (and lactate production) [10,12,13]. Although the effect on organs and tissues other than the liver is less clear, metformin can still diminish mitochondrial respiration and increase glycolysis (and lactate release) in the skeletal muscle [14]. Whether the drug can decrease global O_2 _consumption in either animals or humans remains poorly investigated and unclear [29-31]. Based on these observations, we hypothesize that during metformin or phenformin accumulation, the inhibition of mitochondrial respiration is so strong that the production of lactate (by the liver and, probably, other tissues) increases above the residual capacity of the body to clear it, leading to the development of lactic acidosis.

Our results support this hypothesis. In fact, systemic O_2 _consumption, measured in 11 patients, was markedly depressed in the early phase, when lactic acidosis was more dramatic, despite a normal, or even increased, O_2 _delivery. This finding may be cautiously extended to 13 additional patients in whom systemic O_2 _consumption could not be computed, from initial recording of very low values of arterio-venous difference in O_2 _content, diminished peripheral O_2 _extraction and increased central venous O_2 _saturation. Similar changes occur after exposure to cyanide, a well-known inhibitor of mitochondrial respiration [32]. Even if acidosis was more likely the result of a diminished mitochondrial respiration, it might have also contributed to further decrease the systemic energy expenditure and O_2 _consumption [33]. However, the basal systemic O_2 _consumption of 15 critically ill, mechanically ventilated patients enrolled in a previous trial led by our group, with an arterial pH below 7.20, was 123 ± 65 ml/min/m^2 ^[34].

Alterations in O_2 _consumption were apparently paralleled by changes in CO_2 _production. Direct measurement of systemic CO_2 _production using the reverse Fick equation requires calculation of the whole blood veno-arterial difference in CO_2 _content. This primarily consists of physically dissolved CO_2_, bicarbonate ions and carbamino compounds. As whole blood CO_2 _content is not routinely measured, we computed it using an algorithm that includes the CO_2 _tension, pH, hemoglobin concentration and O_2 _saturation [17]. Similar to arterio-venous difference in O_2 _content, the initially low difference between venous and arterial CO_2 _content is suggestive of diminished CO_2 _production.

Previous studies have demonstrated that severity of illness, use of sedatives and catecholamines, heart rate, body temperature and mechanical ventilation can all affect resting energy expenditure [35,36]. Overall, systemic O_2 _consumption, arterio-venous difference in O_2 _content and veno-arterial difference in CO_2 _content reached their nadir when severity of illness and use of catecholamines were at their highest values. Patient awakening occurred slowly, well after the normalization of O_2 _consumption and related variables. Heart rate and the need for mechanical ventilation did not significantly change over time. A body temperature on hospital admission averaging 34 to 35°C cannot, in isolation, explain the observed 40 to 60% reduction in systemic O_2 _consumption, because O_2 _consumption should diminish by approximately 5 to 6% for every 1°C fall in temperature [37,38]. Moreover, the systemic O_2 _consumption of 25 critically ill patients, with a body temperature between 34 to 35°C, was 136 ± 40 ml/min/m^2 ^[34]. None of the patients included in the present study had any obvious reason to be hypothermic on hospital admission: they usually arrived from home, were awake and with pale, cold extremities. Hypothermia was more likely the consequence of the biguanide-induced decrease in metabolic rate. Even if abnormally low body temperature may impact upon the interpretation of the blood gas analyses performed at 37°C, temperature correction is unnecessary to compute the arterio-venous differences in O_2 _and CO_2 _content [39].

Some of the limitations of this present study deserve a comment. First, we did not include any control group, because of the peculiar characteristics of the study population. However, every single patient with biguanide intoxication acted as an internal control, with individual recordings of global O_2 _consumption (and CO_2 _production) being significantly lower on day 1, relative to the following days. Second, we used the central venous O_2 _saturation to compute global O_2 _consumption of patients equipped with a cardiac output monitoring but not a pulmonary artery catheter. As catecholamine use did not change over time in these subjects, changes in central venous O_2 _saturation (and derived variables) likely reflected those in mixed venous O_2 _saturation. Moreover, when the analysis was restricted to the 7 patients equipped with a pulmonary artery catheter, the major findings of the study remained valid. Third, the respiratory quotient - the ratio between the difference in CO_2 _and O_2 _content of simultaneously drawn arterial and venous blood samples - sometimes exceeded one, an unexpected finding, at least at steady state. Possible explanations include the fact that, in our study population, blood gas analysis were not performed at steady state and blood CO_2 _content was estimated rather than directly measured. We cannot, however, definitely exclude the occurrence of any error in blood sampling, gas analysis or data reporting.

## Conclusions

Metformin and phenformin intoxication is characterized by severe lactic acidosis and abnormally low systemic oxygen consumption despite normal or even increased systemic oxygen delivery. These findings are consistent with the hypothesis that biguanide drugs cause lactic acidosis by inhibiting mitochondrial respiration, without any clear evidence of cellular hypoxia. Cause and effect still needs to be conclusively demonstrated.

## Key messages

• The progressive increase in metformin use may result in a parallel increase in the incidence of associated lactic acidosis.

• The pathogenesis of biguanide-associated lactic acidosis remains unclear, especially when it develops in the absence of other major risk factors.

• Biguanide intoxication is characterized by severe lactic acidosis and abnormally low systemic O_2 _consumption, despite normal or even increased global oxygen delivery.

• Resolution of drug intoxication is paralleled by correction of lactic acidosis and normalization of systemic O_2 _consumption.

• These findings are in line with the hypothesis that lactic acidosis develops during metformin or phenformin intoxication because of inhibition of mitochondrial respiration.

## Abbreviations

C(a-v)O_2_: arterio-venous difference in oxygen content; C(v-a)CO_2_: veno-arterial difference in carbon dioxide content; CaO_2_: arterial blood oxygen content; CvO_2_: venous blood oxygen content; CI: cardiac index; CO_2_: carbon dioxide; DO_2_: systemic oxygen delivery; O_2_: oxygen; OEI: oxygen extraction index; PaO_2_: arterial venous oxygen tensions; PvO_2_: central venous oxygen tensions; RASS: Richmond Agitation Sedation Score; SAPS II: Simplified Acute Physiology Score II; SaO_2_: arterial oxygen saturation; SOFA: Sequential Organ Failure Assessment; SvO_2_: central venous oxygen saturation; VCO_2_: systemic carbon dioxide production; VO_2_: systemic oxygen consumption.

## Competing interests

The authors declare that they have no competing interests.

## Authors' contributions

AP conceived the study, participated in its design and coordination, performed the statistical analysis and drafted the manuscript. RR, PT, and SV participated in study design and data collection. MS, AR, and GF participated in data collection, interpretation of data and helped to draft the manuscript. AR participated in study design and data collection. GM participated in data collection and helped with statistical analysis. LG participated in study design, interpretation of data and helped to draft the manuscript. All the authors read and approved the final manuscript.
